# Detection and genetic characterization of equine viruses in Sweden using viral metagenomics

**DOI:** 10.1186/s12917-025-04613-2

**Published:** 2025-02-27

**Authors:** Anne-Lie Blomström, Annika Källse, Miia Riihimäki

**Affiliations:** 1 Department of Animal Biosciences, Swedish University of Agricultural Sciences, Uppsala, Sweden; 2 Department of Clinical Sciences, Swedish University of Agricultural Sciences, Uppsala, Sweden

**Keywords:** Horses, Fever, Viral metagenomics

## Abstract

**Background:**

Viral infections pose a significant challenge to the equine population, compromising welfare and causing substantial economic losses for the global equine industry. While numerous equine viral pathogens have been identified, many suspected viral infections remain undiagnosed. This highlights the need for further identification and characterization of viruses circulating within the equine population.

In this study, we utilized viral metagenomics to investigate viruses present in serum samples and nasal swabs collected from horses in Sweden. The primary focus was on horses presenting with fever, although control horses were also included for comparison.

**Result:**

The viral metagenomic analysis identified several viruses in the investigated samples. Among nasal swabs, the majority of the viral reads were classified as various equine herpesvirues (EHVs), mainly EHV-2 and EHV-5. Other viruses in nasal swabs include but are not limited to EHV-4, Torque teno equus virus 1 (TTeqV1) and equine copiparvovirus (eqCopV). Both TTeqV1 and eqCopV were also detected in the serum samples together with equine circovirus and equine pegivirus. A number of the detected viruses were further genetically characterized and were shown to display high sequence similarity to viruses from the US and/or China. qPCR screening of a selected number of the detected viruses revealed a rather low detection rate (1.6%–9.4%) in individual horses.

**Conclusion:**

This study identified several viruses that circulate in the horse population in Sweden, some of which have not been previously detected in Sweden or Europe. Furthermore, the complete or nearly complete genomes of several of these viruses have been genetically characterized. These new data provide a valuable foundation for developing improved detection assays and conducting larger prevalence studies to assess the potential impact of these viruses on the equine population. Such efforts could ultimately contribute to enhanced equine welfare.

**Supplementary Information:**

The online version contains supplementary material available at 10.1186/s12917-025-04613-2.

## Background

The global horse population is estimated at approximately 60 million, with 5 million in Europe (FAOSTAT/UNData) and 360 000 in Sweden (The Swedish Board of Agriculture). Like other species, horses face constant threats from viral infections, which can compromise equine welfare and, in some cases, result in significant economic losses for the equine industry worldwide [1, 2]. These infections, caused by various viral agents, can produce a broad spectrum of clinical symptoms and outcomes, ranging from subclinical infections to life-threatening conditions. A wide range of equine viruses, including several well-characterized pathogens, have been described and are routinely included in diagnostic protocols worldwide. Major equine pathogens include equine herpesviruses (EHV-1 and EHV-4), equine influenza virus, West Nile virus, African horse sickness virus, equine viral arteritis virus, equine coronavirus, and equine encephalomyelitis viruses [1–5]. However, many suspected viral infections remain undiagnosed, as these key pathogens are often not identified in clinical cases.

The use of high-throughput sequencing combined with bioinformatic analysis, known as viral metagenomics [6], offers a powerful approach to investigate equine samples from both healthy and diseased animals. Viral metagenomics allows the detection of all viruses present in a sample simultaneously without the need to target any specific virus. It also allows the genetic characterization of both known and previously unknown viruses [7, 8]. This methodology can thus increase our understanding of which viruses are circulating within the global horse population and will support the development of new diagnostic and screening tools, enabling more accurate diagnoses and improving treatment and control measures for equine viral diseases.

At present, few viral metagenomic studies have been conducted on samples derived from horses, the majority of which have been conducted in either the US [9–11] or China [12]. Therefore, as many suspected viral infections are undiagnosed, in this study, we used viral metagenomics to identify viruses in horses with fever in Sweden. The viral metagenomes of both the serum and nasal swabs of these horses were characterized.

## Methods

### Samples

The horses included in this study were owned by private individuals and consent to participate was obtained from each individual owner. Serum samples and unilateral nasal swabs (e-swab (Copan, Brescia, Italy)) were collected at the SLU University Animal Hospital, Uppsala Sweden from 37 horses with high fevers (≥ 38.5°C). Before inclusion in this study, these horses underwent routine diagnostic testing at the animal hospital to determine the cause of fever. As part of this investigation, they were tested for common respiratory viruses (EHV-1, EHV-4, and equine influenza virus) through the horse airway analysis package at the Swedish Veterinary Agency. All tested negative, with the exception of two horses that were positive for EHV-4. The main symptom at testing was fever although a few also presented other symptoms such as diarrhea, colic and respiratory signs. In addition, serum samples and nasal swabs were also collected from 27 nonfebrile horses visiting the clinic for other (noninfectious-related) reasons. An ethical permit (no. 5.8.18–15,528/2021) was obtained from the Swedish Board of Agriculture prior to sampling. All the horses were included in the viral qPCR screening, and 21 were investigated through viral metagenomics.

### Sample preparation and nucleic acid extraction for viral metagenomics

From each horse, 400 μl of serum and 400 μl of nasal swab buffer was taken and filtered through a 0.45 Ultrafree®-MC Centrifugal µm filter (Merck, Darmstadt, Germany) at 12,000 × g for 4 min in order to remove bacteria. The filtrate was divided into two aliquots, one for RNA extraction and one for DNA extraction. In order to reduce host DNA, the DNA aliquot was subjected to nuclease treatment for 1 h at 37°C with 2 μg of RNase (Thermo Fisher Scientific, Waltham, MA, USA) and 10 U of TURBO DNase (Invitrogen, Carlsbad, CA, USA) prior to DNA extraction using GeneJET Genomic DNA Purification Kit (Thermo Fisher Scientific, Waltham, MA, USA) in accordance with the manufacturer’s instructions. A total of 750 μl of TRIzol (Invitrogen, Carlsbad, CA, USA) was added to the RNA aliquot, and the RNA was extracted via a combination of TRIzol and the GeneJET RNA Purification Kit protocol as previously described [13]. The RNase-Free DNase Set (Qiagen, Hilden, Germany) and RNeasy MinElute Cleanup Kit (Qiagen, Hilden, Germany) were used to DNase treat, pool and concentrate the RNA. In order to remove host and bacterial ribosomal RNA (rRNA), the RNA was subjected to rRNA depletion using rRNA depletion Ribo-Zero Plus rRNA Depletion Kit (Illumina, San Diego, CA, USA) in accordance with the manufacturer’s instructions.

### cDNA and DNA labeling

For the rRNA-depleted RNA, during cDNA synthesis, a tag-sequence was incorporated by using the primer FR-20N (GCC GGA GCT CTG CAG ATA TCN NNN NN) [14]. First-strand synthesis was performed using Superscript III (Invitrogen, Carlsbad, CA, USA) according to the manufacturer's instructions. Second-strand synthesis was performed by adding Klenow fragment (3′ → 5′ exo-) (New England Biolabs, Ipswich, MA, USA) to the first-strand cDNA. The second strand synthesis reaction was run for 1 h at 37°C before a 10-min termination step at 75°C. The extracted DNA was labeled during a Klenow fragment (3′ → 5′ exo-) reaction using the same primer and temperature as those used for the cDNA reaction. After the labeling reactions, both samples were treated identically.

### Random PCR (rPCR)

The labeled DNA and cDNA were amplified using the primer FR-26RV (GCC GGA GCT CTG CAG ATA TC) [14] under the following conditions: 1X PCR buffer, 2.5 mM MgCl_2_, 2.5 mM deoxynucleoside triphosphates (dNTP), 0.4 mM FR-26RV primer, and 1.25 U AmpliTaq Gold DNA polymerase (Applied Biosystems, Foster City, CA, USA). The amplification was initiated with a 10-min heating step at 95°C, followed by 40 cycles of 30 s at 95°C, 30 s at 58°C, and 90 s at 72°C and ended with an extra elongation step at 72°C for 10 min. The rPCR products were purified using GeneJET PCR Purification Kit (Thermo Fisher Scientific, Waltham, MA, USA) and subsequently used for library constructions. Prior to sequencing, the rPCR products were pooled so that each pool contained 4–5 horses. Separate pools (four from horses with fever and one from nonfever horses) were made for the nasal and serum samples, respectively.

### Nanopore sequencing

The sequencing libraries were constructed using the Native Barcoding Kit 24 V14 (Oxford Nanopore Technologies, Oxford, UK) in accordance with the manufacturer’s instructions, with a few exceptions, as detailed next. As an input, 250 ng of purified rPCR product was used. The end-repair reaction was run for 15 min at 20°C and 15 min at 65°C, and the native barcode ligation reaction was incubated for 20 min at 20°C and 10 min at 65°C. Libraries were sequenced on flow cells (R10.4.1) for approximately 18 h using live base calling.

### Metagenomic sequence analysis

The base-called reads were uploaded to EPI2ME (https://epi2me.nanoporetech.com) and annotated through the “What is in my pot” (WIMP) workflow. In addition, the reads were quality checked and trimmed using the CLC Genomics Workbench (v24.0) (Qiagen, Hilden, Germany). De novo assembly was performed using the CLC Genomics Workbench, and the created contigs and the unassembled sequences (singletons) were combined into one sequence list. The sequences were annotated via blastx (E value ≤ 0.0001) using Diamond (v 2.0.14) [15]. The diamond results were visualized using Megan7 [16]. The CLC Genomics Workbench was used for mapping reads to specific viral genomes and extracting consensus sequences.

### Genetic characterization

For some of the viruses, metagenomic Nanopore sequencing was complemented with additional sequencing to obtain the complete/near complete genome. In this case, PCR primers (see Table 1s in Supplementary material 1) were designed on the basis of the CLC reference genome alignment to close genome gaps. Platinum SuperFi PCR Master Mix (Invitrogen, Carlsbad, CA, USA) was used for PCR in accordance with the manufacturer’s instructions (see Supplementary material 1 for PCR program details), and the PCR products were purified using GeneJET PCR Purification Kit (Thermo Fisher Scientific, Waltham, MA, USA). The purified PCR products were sequenced using Sanger sequencing at Macrogen Europe. CLC Genomics Workbench was used to trim the Sanger sequences.

### Phylogenetic analysis

Reference sequences were downloaded from GenBank and aligned in CLC Genomic Workbench using MUSCLE [17]. The reference sequences were selected based on species assigned by the International Committee on Taxonomy of Viruses (ICTV) to the relevant genus, as well as the availability of complete equine viral genomes. The alignments were imported into IQ-TREE (v.1.6.12) [18, 19] and maximum likelihood trees were constructed using 1,000 ultrafast bootstrap replicates [20] according to the best substitution model by lowest Bayesian Information Criterion (BIC) score. Interactive Tree of Life (iTOL) v7.0 [21] was used for visualization.

### Quantitative PCR

On the basis of the findings of the viral metagenomic analysis, PCR primers were designed for several of the identified viruses (Table 1). Total nucleic acid was re-extracted from all the samples used in the metagenomic analysis as well as from additional samples using the GeneJET Viral DNA and RNA Purification Kit (Thermo Fisher Scientific, Waltham, MA, USA). iTaq universal SYBR® Green one-step kit and iTaq universal SYBR® Green kit (Bio-Rad, Hercules, CA, USA) were used in accordance with the manufacturer’s instructions. The PCR program was as follows: 50°C for 10 min (RT-step for RNA viruses); 95°C for 2 min followed by 40 cycles of 95°C for 10 s and 60°C for 30 s; and a melt curve step 65°C to 95°C (increment 0.5°C 5 s).

**Table 1:** Viruses targeted in the qPCR analysis, including their primer sequences (5´−3´ direction) and PCR target size

	**Forward**	**Reverse**	**Target size (nt)**
Torque teno equus virus 1 (TTeqV1)	CTCAGGTATTAGAGGAGTAGCT	GTCTGGGATCAGTATATTCTGC	169
Equine circovirus 1 (eqCV1)	TATGGCGCAAACTTATTGGA	CTCAGCAGTACATCCCATG	115
Equine copiparvovirus (eqCopV)	ACAGTAAGACCATTTAGAATGAC	GTCCTGGTAGCCATGTTAC	139
Equine pegivirus (ePgV)	ATTATACCGGTAGCCTGGT	CATGACCTGGTCATCATCAA	158

## Results

### High-throughput sequencing output

The nanopore sequencing yielded approximately 30 million sequence reads in total (Table 2). The read length varied, after quality trimming, between 436 and 527 nucleotides (nt), with an average read length of 495 across all sequencing pools.

**Table 2:** Overview of the nanopore sequencing output; s = serum; n = nasal swab

Pool ID	No. raw reads	No. trimmed reads	Average read length (nt) after trimming	No. singletons/contigs
1s (fever)	3 882 492	3 881 864	469	391 945/71 333
1n (fever)	2 924 010	2 923 340	516	883 712/758 982
2s (fever)	2 350 985	2 350 526	522	481 086/42 119
2n (fever)	2 441 372	2 440 830	511	830 323/710 712
3s (fever)	2 938 779	2 140 794	513	502 455/49 837
3n (fever)	2 465 164	2 464 594	501	843 572/636 789
4s (fever)	2 938 779	2 938 403	527	647 259/60 181
4n (fever)	2 109 702	2 109 307	486	742 023/599 165
5s (nonfebrile)	5 119 080	5 117 986	472	682 696/145 642
5n (nonfebrile)	3 391 269	3 389 992	436	930 299/905 989

### Annotation overview

The initial classification of the individual reads was performed in real time using EPI2ME, and through this analysis, 16–76% of the reads were classified (Table 3). In most pools, the majority of the classified reads belonged to eukaryotes and hence were of host origin. Despite the filtration step, in some of the pools, a high percentage of the classified reads were of bacterial origin. Few reads (< 1%) were classified as archaea, while < 1% to 31% of the reads in the different sequencing pools were classified as viruses.

**Table 3:** Overview of the sequencing read classifications

Pool ID	Unclassified (% of total)	Classified (% of classified)			
		*Eukaryote*	*Viruses*	*Bacteria*	*Archaea*
1s (fever)	24%	22%	1.5%	76%	< 1%
1n (fever)	75%	67%	31%	1.5%	< 1%
2s (fever)	65%	33%	< 1%	66%	< 1%
2n (fever)	82%	93%	< 1%	5.4%	< 1%
3s (fever)	63%	50%	< 1%	50%	< 1%
3n (fever)	77%	73%	< 1%	25%	< 1%
4s (fever)	55%	20%	< 1%	79%	< 1%
4n (fever)	84%	94%	2.4%	3%	< 1%
5s (nonfebrile)	64%	36%	< 1%	63%	< 1%
5n (nonfebrile)	76%	62%	24%	14%	< 1%

To allow more precise annotation and identification of viruses in the different pools, the reads were quality trimmed and de novo assembled, and the contigs as well as the remaining unassembled reads (singletons) (Table 2) were subjected to blastx analysis using Diamond [15, 16]. This analysis revealed the presence of both DNA and RNA eukaryotic viruses (Table 4). Overall, the viral composition varied between the two sample types (nasal swabs vs serum). In nasal swabs, a majority of the viral reads were classified within the family *Orthoherpesviridae*. In addition to *Orthoherpesviridae*, several other DNA viruses have been identified, including those belonging to the families *Anelloviridae*, *Papillomaviridae* and *Parvoviridae*. Few RNA viruses were identified in the nasal swabs, and apart from the retroviruses in pool 5n, all of these were unclassified viruses within the *Riboviria* realm or in the *Picornavirales* order. Additionally, in the serum samples, most of the viral families identified were those with a DNA genome, although RNA viruses were also identified. The DNA viral families identified in the serum include *Anelloviridae* and *Parvoviridae*. Among the RNA viruses, *Flaviviridae* were identified in four out of the five pools. A number of the viruses detected, with a focus on those not previously detected in Sweden, in the nasal swabs and/or in the serum samples were studied in greater detail.

**Table 4:**
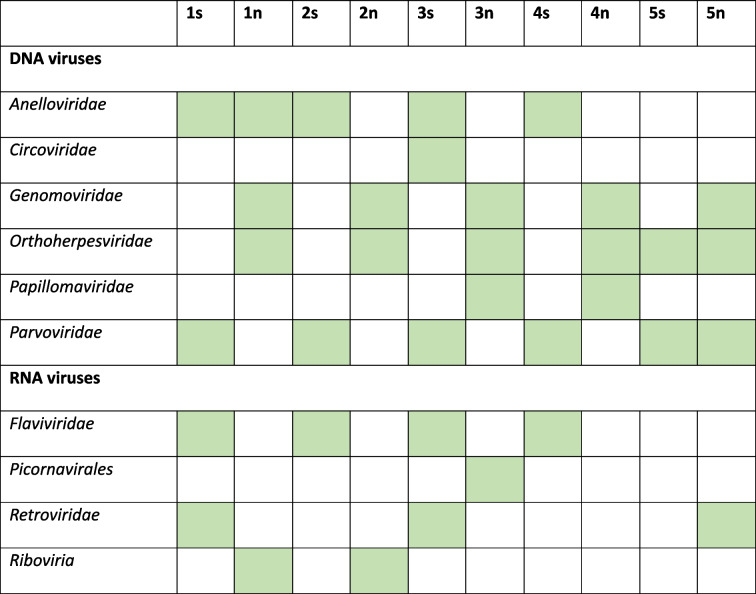
Viral families identified in the serum and nasal swabs pools, including *Riboviria* and *Picornavirales*; green indicates that the virus was identified

### Flaviviridae

**Fig. 1:**
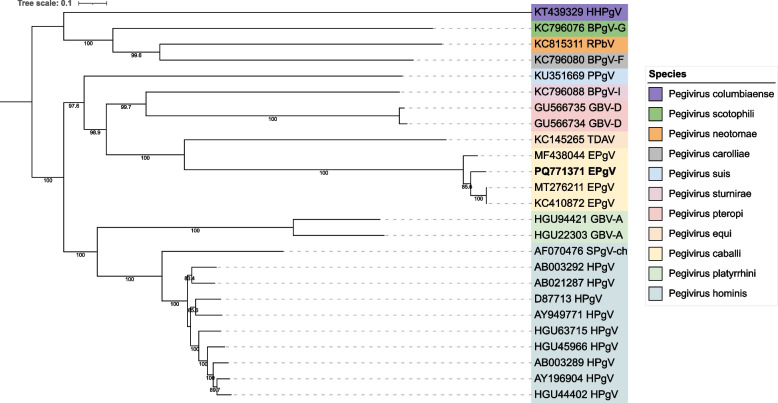
Phylogentic analysis of genus *Pegivirus*. Maximum likelihood tree of representative member species in the genus *Pegivirus*. The Best-fit model (TIM2 + F + I + G4) was chosen according to BIC and bootstrap 1 000 was used. The tree displays bootstrap values ≥ 70 and the color labels represent the different species. The sequence obtained in this study is displayed in bold

Flavivirus sequences were identified in all of the serum pools (1s-4s) from febrile horses but not in those from 5s. The identified viruses were classified into the genus *Pegivirus,* and the sequence reads were dispersed across the genome but did not fully cover it. However, through PCRs covering the gaps and additional sequencing, the complete coding region of the genome was obtained. This sequence (PQ771371) is 10 035 nt in length and encodes a 3 344 amino acid (aa)-long polyprotein. Three complete genomes and four polyprotein sequences are available in GenBank, and the identity to these were 90 – 91% and 98 – 99%, respectively. Phylogentic analysis of the, by ICTV, recognized species as well as the publicly available complete equine pegiviruses (eqPgV) show that the pegivirus identified in this study (PQ771371) cluster with the viral members belonging to species *Pegivirus caballi* (Fig. 1).

### Anelloviridae

Through sequencing, sequences matching Torque teno equus virus 1 (TTeqV1) could be identified in all of the serum pools, with the exception of 5s. In addition, a low number of reads could also be detected in one of the five nasal swabs (Table 4). Sequence analysis revealed that the TTeqV1 identified in these samples belonged to the genus *Mutorquevirus*. Most TTeqV1 reads were identified in pool 1s, and from this pool, a complete genome was obtained. TTeqV1 has not been previously identified outside of the US, and only one complete genome (NC_040668) is available in GenBank. The genome (PQ771369) obtained through this study was 2 197 nt in length and presented high nucleotide sequence similarity (98.6%) to that of NC_040668, indicating that this virus belongs to the species *Mutorquevirus equid1*. For the other member of the genus *Mutorquevirus*, Torque teno equus virus 2 (NC_076942; TTeqV2), 59% nt sequence similarity was observed over the overlapping genome.

### Parvoviridae

**Fig. 2:**
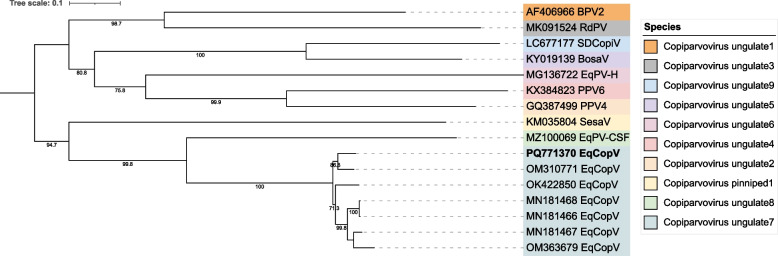
Phylogentic analysis of genus *Copiparvovirus*. Maximum likelihood tree of representative member species in the genus *Copiparvovirus*. The Best-fit model (TIM3 + F + I + G4) was chosen according to BIC and bootstrap 1 000 was used. The tree displays bootstrap values ≥ 70 and the color labels represent the different species. The sequence obtained in this study is displayed in bold

Reads belonging to the family *Parvoviridae,* genus *Copiparvovirus,* were identified in all the serum pools, including one nasal swab pool (5n). The complete coding genome sequence (PQ771370) was obtained. This sequence was 4 965 nt long and encodes two open reading frames (ORFs), NS1 (585 aa) and VP2 (1 065 aa). Six complete equine copiparvovirus genomes are available in GenBank, and a 92 – 94% nt sequence similarity to these sequences was observed. The NS1 amino acid identity to other equine copiparvoviruses, including to the isolate EqCoPV_8 (MN181466) classified by ICTV to the species *Copiparvovirus ungulate7*, were 94—98%. This together with the phylogenetic analysis (Fig. 2) shows that the characterized equine copiparvovirus (eqCopV) belongs to the species *Copiparvovirus ungulate7*.

### Circoviridae

In pool 3s, 10 contigs matching equine circovirus 1 (EqCV1) were identified. The complete coding region for the capsid protein was obtained (PQ771368) and compared to only EqCV1 (EqCV1-Charaf; NC_077115), which is available in GenBank. The two EqCV1 capsid genes/proteins had 98.56% nt identity and 100% aa identity to each other.

### Orthoherpesviridae

In nasal swabs, the majority of the viral reads belong to the family *Orthoherpesviridae* and the genus *Percavirus*. More specifically, the equine herpesviruses (EHVs) identified in this genus were the gammaherpesviruses EHV-2 and EHV-5. Out of these, EHV-2 reads were most abundant in all pools. In one pool (4n), reads belonging to the genus *Varicellovirus* were also identified in addition to *Percavirus*. As for EHV-2 and EHV-5, no complete EHV-4 genome was obtained, and the aa identity of the individual sequence reads to their top Diamond hit varied.

### Other viruses

In the Riboviria group of pool 1n, a few picornavirus reads were found. The majority of these classified as Erbovirus A (also known as equine rhinitis B virus). Unfortunately, few sequences were identified, and no complete or large parts of the genome could be obtained. In pool 3s, different picornavirus sequences classified in the order *Picornavirales* but were not assigned to a specific family. These divergent picornavirus sequences matched to various picornaviruses of different hosts. Unfortunately, no genome could be obtained from these reads.

### qPCR screening

As TTeqV1, eqPgV, eqCopV and eqCV1 have not previously been detected in Sweden or Europe, qPCR systems were set up to enable screening of individual samples. A screening of the 21 samples used in metagenomics as well as samples from an additional 53 horses revealed that few virus-positive serum samples (1.6%—9.4%) were obtained (Table 5). In all of the cases, with the exception of two serum samples, only one virus was identified per sample. In the serum samples with a co-infection, TTVeqV1 and eqCV1, as well as eqPgV and eqCopV, respectively, were identified.

**Table 5:** Percentage of virus-positive samples; n/a = not run

	**Serum**			**Nasal swabs**		
	*Overall*	*Fever*	*Control*	*Overall*	*Fever*	*Control*
**TTVeqV1**	6.3	8.1	3.7	7.8	13.5	0
**eqPgV**	4.7	2.7	7.4	n/a	n/a	n/a
**eqCopV**	9.4	5.4	14.8	n/a	n/a	n/a
**eqCV1**	1.6	2.7	0	n/a	n/a	n/a

## Discussion

Viral infections in horses pose a threat to animal welfare and can lead to economic losses for the horse owner and equine industry [1, 2]. As fever is one of the first signs of a potential acute viral infection, in this study, we used viral metagenomics to identify and, in some cases, genetically characterize viruses in horses with fever.

A large portion of the identified viruses in both the serum and nasal swabs were DNA viruses. In the nasal swabs, EHV-2 and EHV-5 were the most commonly and abundantly detected viruses. The detection of these viruses is unsurprising, as they have a global distribution [2, 22–24], including in Sweden. Neither EHV-2 nor EHV-5 has been confidently linked to disease in horses, as they are also commonly found in healthy horses [25]. EHV-4, which was detected in one of the pools, is an economically important virus known to cause respiratory diseases, including fever [4]. This EHV-4 positive sequencing pool also contained the two horses that prior to entering this study had in the routine diagnostic tested positive for EHV-4.

Apart from herpesviruses, TTVeqV1 was also identified in nasal swabs. In addition, TTVeqV1 was detected in the serum samples of horses with fever. TTVeqV1 is a virus with a single-stranded circular DNA genome that was first identified and genetically characterized from a plasma sample collected in 2012 from a horse in the US [10]. There is only one complete genome publicly available for this virus (NC_040668) and one additional genome for TTVeqV2 (NC_076942), the second member of the genus *Mutorquevirus* [26]. Our genome (PQ771369) showed high sequence similarity (99%) to the TTVeqV1 genome from the US, indicating high genetic conservation both geographically and over time. The potential role of torque teno viruses in equines remains unknown, as very limited data to no data is available regarding genetics, prevalence, and potential pathogenicity.

EqCV1, another single-stranded circular DNA virus, but a member of the family *Circoviridae*, was detected in the serum of one of the horses with fever. EqCV1 was discovered for the first time in the serum of a horse in the US that displayed hepatitis and fever [27]. The capsid protein of the US eqCV1 and the protein discovered in this study presented high sequence similarity at both the amino acid and nucleotide sequence levels. These are the only two equine circoviruses available in GenBank, and they are most closely related to a Canadian elk circovirus [28] as well as to porcine circoviruses (PCVs). As for equine TTVs, no investigations have been conducted regarding the potential role of equine circoviruses in horse welfare. However, circoviruses have been extensively investigated in pigs, where PCV-2 is widespread and recognized as a significant pathogen associated with several disease complexes [29]. Several studies have explored the potential effects of coinfection with PCV-2 and porcine TTVs [14, 30–33], although no definitive conclusions have been reached. This is particularly relevant here, as one of the horses was found to have a coinfection of eqCV1 and TTeqV1.

An additional single-stranded DNA virus but belonging to the family *Parvoviridae* and genus *Copiparvovirus*, eqCopV, was also detected and genetically characterized for the first time in Sweden and Europe. This virus was first found in a horse with respiratory signs in the US [9], and with extended screening, it could be identified in several horses, including additional horses with respiratory signs but also in healthy control horses [34]. In addition to the US, eqCopV has also been detected in China [35] and South Korea [36]. In our study, 6 out of 64 samples (9.4%) were positive. The positive samples originated from both febrile and nonfebrile horses. In the US study, eqCopV DNA was detected in 20.5% of the investigated horses [34]; in the Chinese study, it was detected in 0.9% of the samples [35]; and in the South Korean study, it was detected in 9.8% [36]. On the basis of these studies, no associations with disease have been made.

The main RNA virus identified in the serum was eqPgV, which belongs to the family *Flaviviridae* and genus *Pegivirus*. The complete coding region of the genome was characterized and showed a 90–91% nucleotide identity to the three available genomes, two from the US [37, 38] and one from China [39]. At present, no association with disease has been made in any of these countries.

## Conclusion

In summary, while our metagenomic study identified several viruses, further investigations using larger sample sets are necessary to determine whether any of these viruses contribute to disease in horses, either individually or through coinfections. Many of the detected viruses are commonly found in other species, often regardless of disease status.

Nonetheless, this study expands our understanding of these viruses by providing additional genetic information, which is often scarce, with only one or two genomes available for some viruses. Moreover, most of these viruses have previously been studied only in the United States and Asia, making this the first detection of several of them in Europe.

## Supplementary Information

Supplementary Material 1.

## Data Availability

The raw sequence reads from the Nanopore sequencing were uploaded to the SRA database under the Bioproject PRJNA1198089: https://www.ncbi.nlm.nih.gov/bioproject/?term=PRJNA1198089. The characterized viral genome sequences have been assigned the NCBI GenBank accession numbers PQ771368-PQ771371.
