# Supplementary material for: Detection and genetic characterization of equine viruses in Sweden using viral metagenomics
Source: BMC Vet Res. 2025 Feb 27;21:119. doi: 10.1186/s12917-025-04613-2 (PMC11866639; doi:10.1186/s12917-025-04613-2)
Supplement: Supplementary file 1 — Supplementary Material 1. [file 12917_2025_4613_MOESM1_ESM.docx]

Supplementary Material 1.

**Suppl. Table 1s** Primers used for viral genetic characterization

| **Primer name** | **Primer sequence (5´-3´direction)** |
| --- | --- |
| F_eqTTV_1726 | TCAGAGAGCATTACTTCAGC |
| R_eqTTV_33 | GTACCAACGACGTCTGTTC |
| F_eqcopi_1478 | TGGAGATACTGTGCATACAC |
| R_eqcopi_2650 | CTCCGGTAGACCAATGTC |
| F_eqcopi_1 | ATGACAGAGAGATTCTTCACAG |
| R_egcopi_635 | AGCCTTTCTGAGTAGTCCT |
| F_eqcopi_735 | GGCTAACAGACAATGCCAT |
| R_eqcopi_1171 | GAAATGGGAAGTGTTCACTG |
| R_eqcopi_end | TTATCTCATTCTTCTGGGTCTG |
| F_eqcopi_4217 | GAACAGAAGAACCAGTAGTACT |
| F_eqcirco_570 | CCAAGTCCACGTCATCAT |
| R_eqcirco_355 | CCTTATTTTGCAGGTCAGTG |
| R_eqcirco_1133 | CACACTCTATGTACAATTCAGG |
| F_eqcirco_1077 | ATTAAACTTCATTGGGGTCTTG |
| F_eqpegi_start500 | ATGCTGCAGCGCAGGCG |
| R_eqpegi_1642 | ATTGGATGGCACGCAGATGA |
| F_eqpegi_2171 | GGACTGCTCTGTCAATACTG |
| R_eqpegi_5540 | CGGCTCAACCACTACTTC |
| F_eqpegi_5722 | AATGTTTCCCCAGCTATGG |
| R_eqpegi_6837 | TGAACACAGAACACTAGTGC |
| F_eqpegi_6999 | ATGCTTCACCATCACGAC |
| R_eqpegi_7749 | GCAGGTGTACTGAGGAATC |
| F_eqpegi_8245 | ATCATTGTCGTGAGTTCTCG |
| F_eqpegi_9677 | ATGATGAGGCAGTCATCTC |
| R_eqpegi_end10533 | TCACACTAGGGCCGCAAG |

PCR program for the virus genetic characterization was as follows: 98°C 2 min; 40 cycles of 98°C 10 sec, 60°C 10 sec and 72°C 15 sec/kb; 72°C 5 min
